# Supplementary material for: Optimizing video data security: A hybrid MAES-ECC encryption technique for efficient internet transmission
Source: PLoS One. 2024 Nov 22;19(11):e0311765. doi: 10.1371/journal.pone.0311765 (PMC11584095; doi:10.1371/journal.pone.0311765)
Supplement: Attachment — Submitted filename: comments.docx [file pone.0311765.s001.docx]

The paper proposes a hybrid technique for video encryption and decryption, combining the advantages of the Modified Advanced Encryption Standard (MAES) and Elliptic Curve Cryptography (ECC) algorithms. The proposed technique encrypts each video frame using MAES and then encrypts the encrypted frames using ECC. The same process is performed in reverse order for decryption. Experimental results show that the proposed technique provides higher security, better accuracy, and shorter processing times compared to other well-known techniques. However, the paper need following minor changes for improvement of paper quality for publication:

Comment 1: To ensure the highest standards of academic communication, I recommend that the manuscript undergo a thorough review. This step is crucial not only to eliminate grammatical errors but also to enhance the logical flow of content, thereby improving readability and ensuring that the manuscript effectively communicates its significant contributions.

Comment 2: The figures and tables citations require substantial improvement in their descriptions and all figures should be cited properly in the main text.
