# Supplementary material for: Optimizing video data security: A hybrid MAES-ECC encryption technique for efficient internet transmission
Source: PLoS One. 2024 Nov 22;19(11):e0311765. doi: 10.1371/journal.pone.0311765 (PMC11584095; doi:10.1371/journal.pone.0311765)
Supplement: Attachment — Submitted filename: Response to reviewer PLOS one.docx [file pone.0311765.s002.docx]

**Reviewer 1 Comments**

**Comment 1:** A more in-depth discussion on the specific modifications made to the MAES and ECC algorithms for hybrid application would be beneficial. This discussion should encompass any optimizations or alterations aimed at enhancing performance for video data encryption. Clarifying the rationale behind key decisions, such as the selection of key sizes and the number of encryption rounds tailored for video data, would provide readers with deeper insights into the design and implementation choices.

**Response 1:** Thanks for the editor feedback and honor the expertise in this domain. By elucidating the rationale behind selecting specific key sizes and encryption round numbers, readers gain a comprehensive understanding of the factors considered during the design process. Justifying these choices involves discussing factors such as the desired level of security, computational efficiency, and the characteristics of video data, which directly impact the encryption process. Through such explanations, readers gain insights into the reasoning behind the decisions made, enabling them to appreciate the thoughtfulness and considerations involved in the design and implementation of the encryption scheme.

**Comment 2:** To ensure the highest standards of academic communication, I recommend that the manuscript undergo a thorough review by a professional editor or a third party. This step is crucial not only to eliminate grammatical errors but also to enhance the logical flow of content, thereby improving readability and ensuring that the manuscript effectively communicates its significant contributions.

**Response 2:** Thank you for your suggestions and comments. The main manuscript has overcome the grammatical mistakes.

**Reviewer 2 Comments**

**Comment 1:** The figures and tables citations require substantial improvement in their descriptions and all figures should be cited properly in the main text.

**Response 1:** Thank you for the valuable suggestion. The suggestion is addressed in the manuscript file and updated.
